# Entangled effects of allelic and clonal (genotypic) richness in the resistance and resilience of experimental populations of the seagrass *Zostera noltii* to diatom invasion

**DOI:** 10.1186/1472-6785-13-39

**Published:** 2013-10-23

**Authors:** Sónia I Massa, Cristina M Paulino, Ester A Serrão, Carlos M Duarte, Sophie Arnaud-Haond

**Affiliations:** 1 CCMAR-CIMAR, Universidade do Algarve, Gambelas, Faro 8005-139, Portugal; 2 Department of Global Change Research, IMEDEA (CSIC-UIB) Institut Mediterrani d’Estudis Avançats, C/Miguel Marqués 21, Esporles, Mallorca 07190, Spain; 3 The UWA Oceans Institute and School of Plant Biology, University of Western Australia, 35 Stirling Highway, Crawley, WA, 6009, Australia; 4 IFREMER, Bd Jean Monnet, BP 171, Sète 34203, France

**Keywords:** Allelic richness, Genotypic richness, Resistance, Resilience, *Zostera noltii*, Biotic stress, Seagrass

## Abstract

**Background:**

The relationship between species diversity and components of ecosystem stability has been extensively studied, whilst the influence of the genetic component of biodiversity remains poorly understood. Here we manipulated both genotypic and allelic richness of the seagrass *Zostera noltii*, in order to explore their respective influences on the resistance of the experimental population to stress. Thus far intra-specific diversity was seldom taken into account in management plans, and restoration actions showed very low success. Information is therefore needed to understand the factors affecting resistance and resilience of populations.

**Results:**

Our results show a positive influence of both allelic and genotypic richness on the resistance of meadows to environmental perturbations. They also show that at the low genotypic (i.e. clonal) richness levels used in prior experimental approaches, the effects of genotypic and allelic richness could not be disentangled and allelic richness was a likely hidden treatment explaining at least part of the effects hitherto attributed to genotypic richness.

**Conclusions:**

Altogether, these results emphasize the need to acknowledge and take into account the interdependency of both genotypic and allelic richness in experimental designs attempting to estimate their importance alone or in combination. A positive influence of allelic richness on resistance to perturbations, and of allelic richness combined with genotypic richness on the recovery (resilience) of the experimental populations is supported by differential mortality. These results, on the key species structuring of one of the most threatened coastal ecosystem worldwide, seagrass meadows, support the need to better take into account the distinct compartments of clonal and genetic diversity in management strategies, and in possible restoration plans in the future.

## Background

The relationship between diversity and ecosystem stability has been explored for a long time in biology, as the number and type of species was expected to determine the specific traits of the ecosystem [1]. Yet the predicted positive correlation between diversity and population/ecosystem stability is still subject to debate [2] as empirical evidence does not universally support it. However, most studies point towards some positive, but variable, effects of higher diversity on stability [3-13]. Recently, the debate on the relationship between diversity and ecosystem stability has intensified, fuelled by concern about the consequences of worldwide biodiversity loss on ecosystems [3,14-21].

Resolving the relationship between biodiversity and ecosystem function is not simple because several components of biodiversity can affect ecosystem functioning, even when considering only diversity at the species level. These components include species richness (the number of species), species evenness (their relative abundance), species composition (their taxonomic or functional nature) and non-additive effects (their interactions) as well as the spatial and temporal variations of those patterns [22]. Stability also has two components: resistance, the ability to withstand disturbance, and resilience, the ability to recover back to an equilibrium state after disturbance [23]. A number of studies have concluded that greater biodiversity (i.e. species richness) can be beneficial. It enhances the stability of communities, the stability or productivity of ecosystems [14,21,24-30], and the resistance to disturbances such as disease and invasion [31,32] or drought [33]. In a slightly parallel line of work, functional richness (number of different plant functional types) and composition has also been shown to increase stability [26,34-41].

In ecosystems where the physical habitat is shaped by one key-species, the genetic diversity within populations of this structural species may have similar importance as species richness does on ecosystem resilience and resistance. Genetic diversity is one of the three main components of biodiversity recognized by the Convention for Biodiversity as a priority target for conservation measures, yet it is still largely neglected in management plans [42]. Genetic diversity is thought to reflect the evolutionary potential of species, as the genome encodes the information necessary to survive and reproduce in the current environment. It also encodes the potential to adapt to changing or alternative environments [43,44]. In strongly declining and threatened populations with critically depleted genetic diversity, both reduced adaptive potential and the possible fixation of deleterious alleles by genetic drift due to small effective population sizes can affect the long term survival capacity of populations and species [45]. Some empirical studies have shown that the genetic composition of key plant populations can have a strong effect at the level of the community and ecosystem [46,47]. It can even enhance diversity of associated species [48]. Yet, empirical evidence gathered thus far to demonstrate the general influence of this component of biodiversity on resistance and resilience of populations or ecosystems arose more recently and from a much more limited number of studies, which may explain its widespread neglect in most management plans thus far.

Ecosystems dominated by one or a few species, such as seagrass meadows or algae stands, are particularly vulnerable, because the loss of genetic diversity resulting from population decline or fragmentation in key-species [49] may have extended consequences on the overall biodiversity and function of the community [50,51]. The presence of seagrasses decreases hydrodynamics and favours the stabilization of the sediment, producing a sheltering habitat for many aquatic species [52]. Seagrass meadows also have a carbon-sink function and estimates suggest they represent between 4.2 and 8.4 billion tons of carbon [53,54]. These essential and emblematic ecosystems are however threatened and declining worldwide [55], and restoration actions taken thus far have shown very low rate of success [56]. Elucidating both the extrinsic and intrinsic factors influencing their decline or resistance, including the genetic components, is a priority.

Recent experimental studies on the seagrass species *Zostera marina*[50,51,57] suggested the importance of genotypic richness, understood as the number of clonal lineages reflecting the number of “distinct genetic individuals”, or genets (Table 1) in clonal organisms [58], on the resistance or resilience to perturbations in stands of this species. Similar results were observed at fine-grained scale in natural stands [59]. Yet the combined influence of genetic diversity *sensu stricto* (i.e. heterozygosity or allelic richness), that can also be designated as genomic diversity [60] was not specifically tested for in all such studies. A lack of correlation between heterozygosity and genotypic richness was put forward when reviewing previous studies [51] to interpret the results as a non-confounded effect of genotypic richness *per se*. However, the associated level of allelic richness, a genetic diversity parameter more sensitive to demographic events [61,62] and more prone to influence the adaptive capacity of populations [61,63], was not disclosed in previous studies that reported positive effects of genotypic richness on stability parameters. Yet, these reports are widely cited using the terms genotypic richness and genetic diversity interchangeably although the correlation between genotypic and genome based (such as allelic richness) measures of diversities were not tested for in the wild [60]. In fact, a recent study based on both simulated and observational data reported that such a correlation, when it exists, is limited to extremely low levels of genotypic richness comparable to those manipulated experimentally, but seldom found in natural populations [60]. Besides, contrasting results reported in natural meadows of *Posidonia oceanica*[49,64] suggest an inverse relationship of stability with genotypic richness and a possible positive influence of genetic diversity (estimated through allelic richness and heterozygosity). Hence, further research on the importance of genetic diversity *sensu stricto* on the stability of seagrass meadows, should attempt to clarify the respective effects of both components by dissociating them. In this study, we tested experimentally the relationship between genetic diversity and the stability of experimental assemblages of *Zostera noltii*, a key-species structuring the intertidal ecosystem of Ria Formosa, to i) test for the level of interdependency of genotypic and allelic richness and ii) test for their respective or combined influence on the resistance and potential for recovery (resilience) of experimental populations.

**Table 1:** Glossary of terms used in this experiment study

**Clonal lineage, or genet**	A set of ramets issued from a single same event of sexual reproduction and resulting from the clonal growth of the single seed or zygote issued from this recombination event. Ramets belonging to the same clonal lineage bear the same multilocus genotype (MLG) or, if somatic mutations have occurred, ramets belong to the same multi-locus lineage (MLL)
**Genotypic richness (= clonal richness)**	Total number of distinct clones (MLGs or MLLs) within each experimental subplot
**Genetic diversity**	The genetic component of diversity. Estimates for genetic diversity can be allelic richness, the total number of alleles, or heterozygosity (unbiased). Here we focus on the estimate of **genetic richness** through **Allelic richness** Â as the total number of alleles within each experimental subplot

## Results

**Figure 1:**
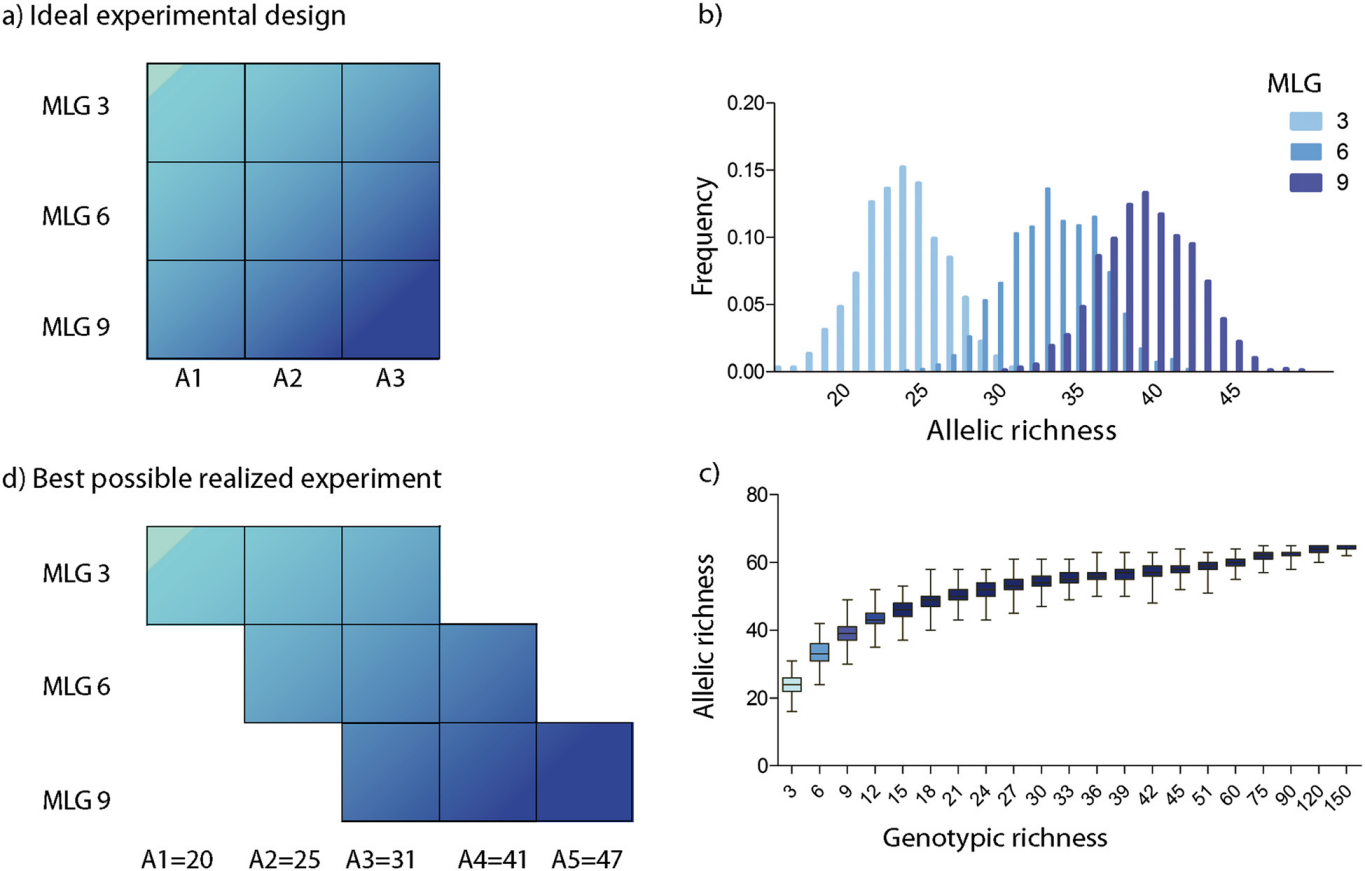
**Ideal vs. possible experimental designs.** 1**a)** Ideal experimental design for each of the 4 plots containing 9 subplots of 27 shoots, with crossed levels of genotypic and allelic richness designed to disentangle their respective effects. **b)** Frequency distribution of allelic richness (in total number of alleles) across the three levels of genotypic richness (3 MLGs in light grey, 6 MLGs in medium grey and 9 MLGs in dark grey). **c)** Evolution of allelic richness for a broader range of levels of genotypic richness. **d)** Best possible design at the levels of genotypic richness manipulated in the experiments, illustrating the composition of each of the 4 plots made of 9 subplots of 27 shoots each, in terms of allelic richness with increasing levels of genotypic richness. The schemes of plot are designed with nested and increasing orders of richness for illustrative proposes, but the positions of the 36 sub-plots in the aquaculture tank where the experiment took place were randomized.

**Figure 2:**
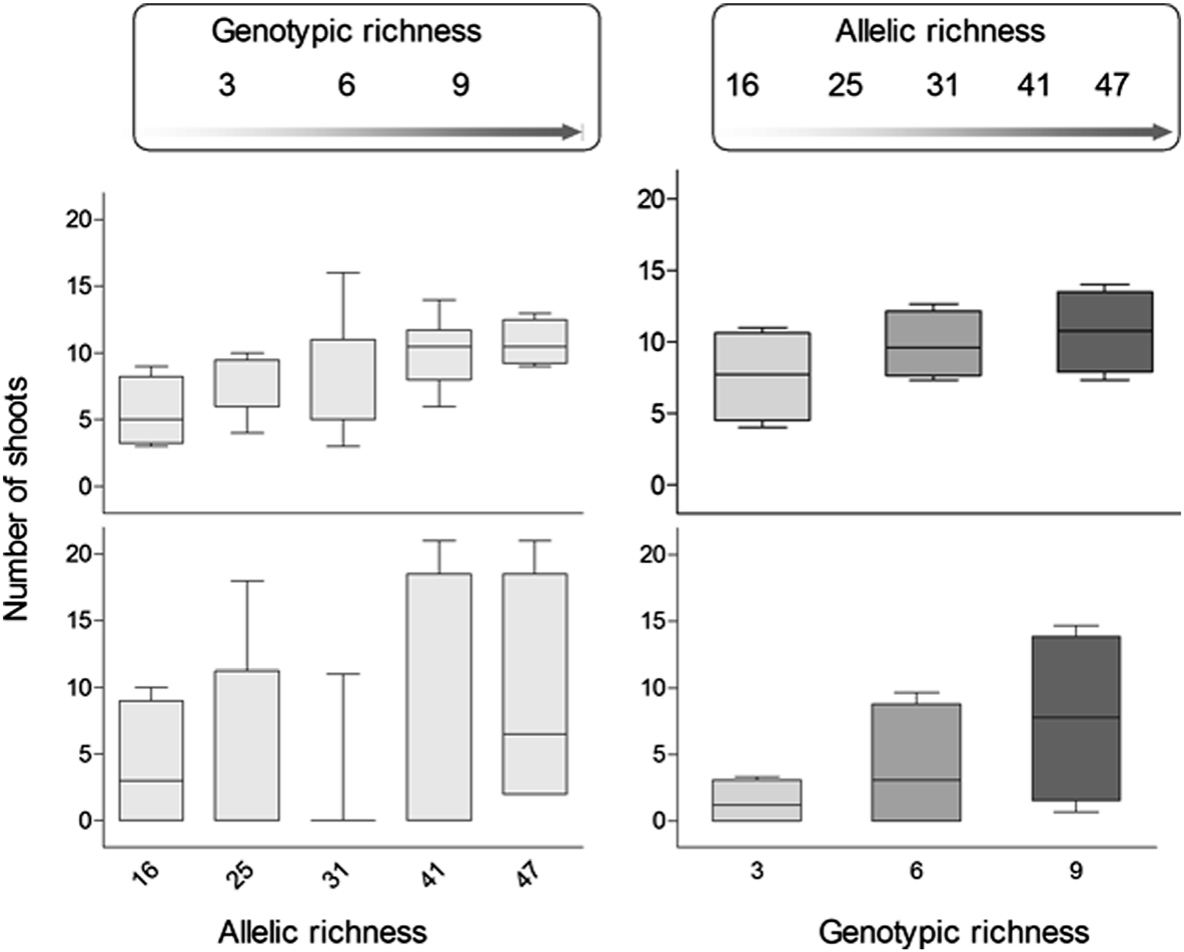
**Evolution of allelic and genotypic richness with time.** Boxplots illustrating the relationships between both allelic (left) and genotypic (right) richness and the number of surviving shoots, after the diatom bloom (top, resistance) and after 10 months survey (bottom, resilience). These graphs illustrate the tendency that could be misleadingly attributed to each parameter alone if ignoring the parallel increase of the other (“hidden effect” illustrated in the upper rectangles with arrows). In regression analysis associated to those graphs, a correlation would be detected between each estimator of richness and the resistance of subplots (upper part of the graphs; p = 0.002 for allelic richness and p = 0.015 for genotypic richness), and only the “genotypic richness” analysis would show a positive relationship with resilience (bottom part of the graphs; p = 0.171 for allelic richness, p = 0.025 for genotypic richness).

Genotyping of the 376 collected clones returned a total of 343 individuals, fully-genotyped at all loci, of which 164 were distinct MLGs. Allelic richness in one thousand possible combinations of 3, 6 and 9 genotypes ranged from 16 to 31 at G = 3, 24 to 42 at G = 6, and 31 to 48 at G = 9 (Figure 1d), with hardly any overlap between the minimum and the maximum levels (Figure 1b, c). A strong correlation between genotypic and allelic richness was observed at the lower levels of genotypic richness, between 1 and 20 (Figure 1b, c), representative of levels commonly manipulated in experiments (r = 0.904, p < 0.001), although it became marginal at higher levels of genotypic richness more typically observed in natural meadows (G > 20). Across 1000 combinations, Â was 23.93 ± 2.63 for G = 3, 33.44 ± 2.97 for G = 6 and 39.39 ± 2.96 for G = 9, and specific low, medium and high levels of allelic richness had to be defined independently for each MLG level. As a result, identical levels of allelic richness could not be standardized for the three genotypic richness levels, and a fractional factorial design was obtained with five levels corresponding to 16, 25, 31, 41 and 47 alleles to distribute among low, medium and high levels of genotypic richness as detailed in Table 2 (Figure 1d). These levels were therefore not equivalent among genotypic richness plots. As an example, the highest level of allelic richness in plots with three genotypes was the same as the intermediate level for 6 genotypes and the lowest level for 9 genotypes (Figure 1d). There was a clear increase in allelic richness levels parallel to the increase in genotypic richness (Figures 1, 2). As a consequence, combined effects of allelic and genotypic diversities could not be simply disentangled through a two-way ANOVA analysis.

**Table 2:** Total number of alleles in each possible combination of genotypic and allelic richness in the experimental design

		**Genotypic richness**		
		**3 MLGs**	**6 MLGs**	**9 MLGs**
	**Minimum**	16	25	31
Allelic Richness	**Medium**	25	31	41
	**Maximum**	31	41	47

Four plots were set with this design, each cell in the table corresponds to sub-plots (each sub-plot is a vase with 27 shoots) with the crossed-level of genotypic and allelic richness. All 36 vases (sub-plots) were randomly arranged in a single aquaculture tank.

The first shoot count, corresponding to a measure of resistance, taken approximately 40 days after the diatom bloom, ranged from 2 to 15, indicating a high but variable differential mortality despite all tanks having appeared to have been covered by similar amounts of green algae. The mean number of shoots increased for both increasing MLG (7.67 for G = 3, 9.75 for G = 6 and 10.75 for G = 9, n = 9) and increasing A (7.5 when A = 16, n = 4; 7.625 when A = 25, n = 8; 9.25 when A = 31, n = 12; 10.75 when A = 41, n = 8; 12.5 when A = 47, n = 4). When analysing the influence of genotypic richness while ignoring the parallel increasing levels of allelic richness (i.e. merging within each MLG class all levels of Â), results supported a significant effect (p = 0.044, Figure 2) on resistance of experimental populations, whereas no significant effect was observed on resilience, as measured 10 months after stress (p > 0.05). Similarly, the effect of allelic richness was significant when ignoring the parallel increase in G (p = 0.028, Figure 2), and the same analysis made on shoot number counted 10 months after the algal bloom did not show any significant trend (p > 0.05). Ignoring one of those parameters would therefore point to the influence, alone, of the other (Figure 2), yet it is clear that higher MLG plots are also bear higher allelic richness and these significant relationships with the number of surviving shoots may be due to either parameter, or to the combination of both.

**Figure 3:**
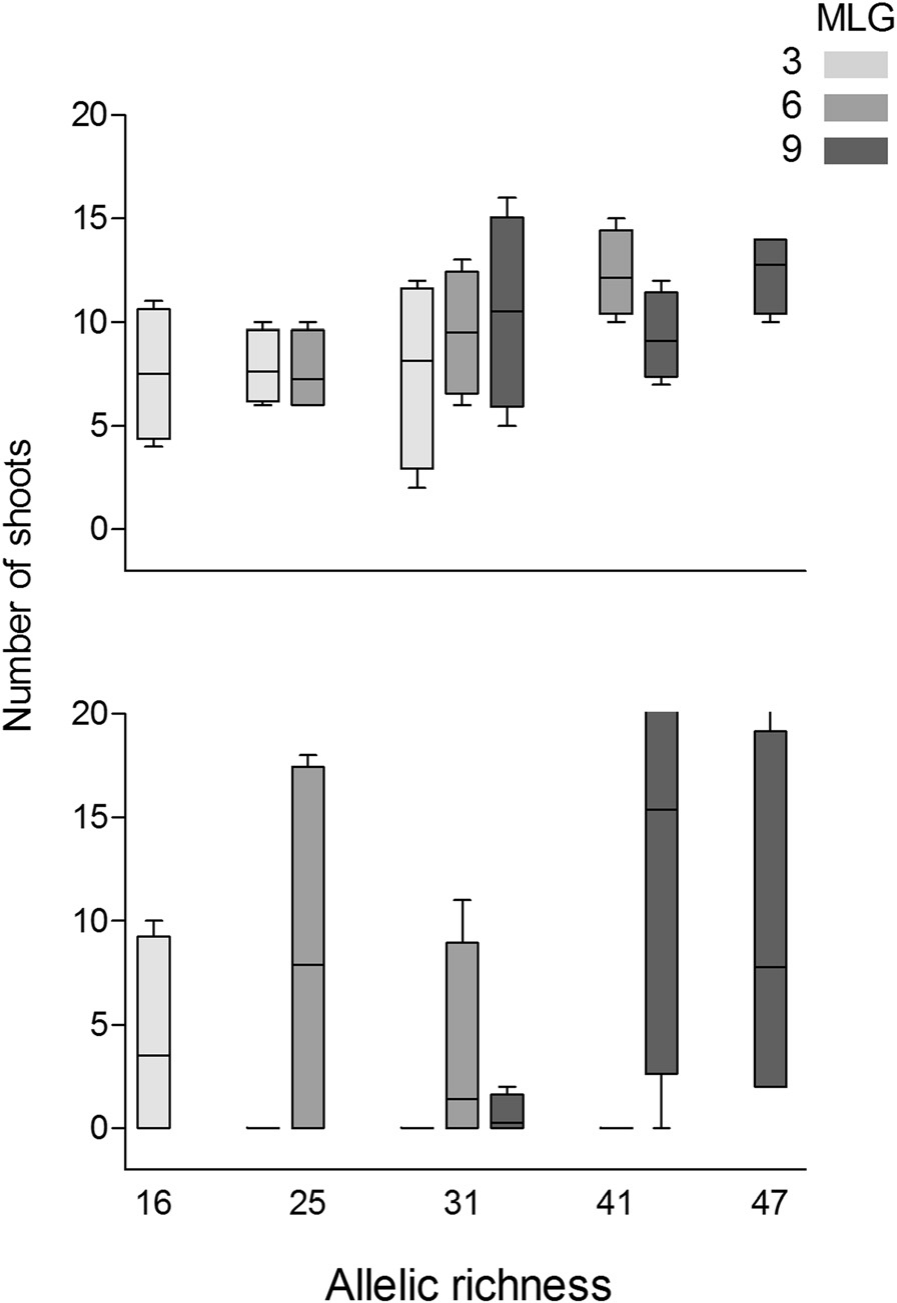
**Combined effect of allelic and genotypic richness on survival.** Mean shoot density for the five levels of allelic richness (16, 25, 31, 41 and 47) in the three genotypic richness levels (3 MLGs in light gray, 6 MLGs in medium-dark gray and 9 MLGs in dark gray). Top: for the first count. Bottom: for the last count. All values are represented by 25th and 75th percentile and minimum and maximum values.

In fact, when the effect of genotypic richness was compared at identical levels of allelic richness among them (specifically Â = 25 for MLG 3 and 6, Â = 31 for all MLG and Â = 41 for MLG 6 and 9; Table 3; Figures 1, 3), the only significant effect (corresponding to Â =41; p = 0.034) showed an inverse relationship with higher genotypic richness inducing lower survival (Table 3). No significant effect of increasing allelic richness was observed either on survival or recovery within any of the three levels of genotypic richness tested individually (Table 3).

**Table 3:** Summary of statistical analysis results

**Source of variation**	**Degrees of freedom**	**P value**
		
*Allelic richness*		
*One-way ANOVA of allelic richness for each genotypic richness level individually (first count)*		
3 MLG	2	0.992
6 MLG	2	0.051
9 MLG	2	0.362
*One-way ANOVA of allelic richness for each genotypic richness level individually (last count)*		
3 MLG	2	0.123
6 MLG	2	0.232
9 MLG	2	0.097
*Genotypic richness*		
*One-way ANOVA for A = 31*	* First count (resistance)*	
R	2	0.629
*One-way ANOVA for A = 31*	* Last count (resistance)*	
R	2	0.469
*t-Test: Paired Two Sample for Means (for identical A)*	* First count (resistance)*	
3 and 6 MLGs	3	0.624
6 and 9 MLGs	3	**0.035**
*t-Test: Paired Two Sample for Means (for identical A)*	* Last count (resistance)*	
3 and 6 MLGs	3	0.066
6 and 9 MLGs	3	0.184
*Multiple regressions*		
*Backward stepwise regression for shoot density (first count - resistance)*		
	32	
Intercept		**0.027**
A		**0.002**
G		-
G*A		-
*Backward stepwise regression for shoot density (last count - resilience)*		
	32	
Intercept		0.905
A		-
G		-
G*A		**0.020**

Values in bold correspond to significant p-value associated to a q-value < 0.05 after the correction for multiple tests. * refers to the interaction of the parameters.

The exploration of the possible effect of Â on the global, wider range of values represented across all plots (Figure 3) by simple regression analysis however showed a highly significant (p = 0.007) overall relationship between allelic richness and resistance in terms of shoot density at 40 days after stress. No significance was observed for resilience, measured as the number of shoots 10 months after the algal bloom (p = 0.368). Similarly, stepwise multiple regression model also showed a significant, positive effect of allelic richness, but no influence of genotypic richness (p > 0.05) or interaction between A and G, on survival measured 40 days after stress. The positive effect of allelic richness on survival was particularly strong following the diatom bloom (p = 0.002, adjusted r^2^ = 0.22, Table 3, Figure 3), and only the interaction between allelic and genotypic richness showed a weak but significant relationship with the density of plots after 10 months recovery (p = 0.020, adjusted r^2^ = 0.13; Table 3, Figure 3).

**Figure 4:**
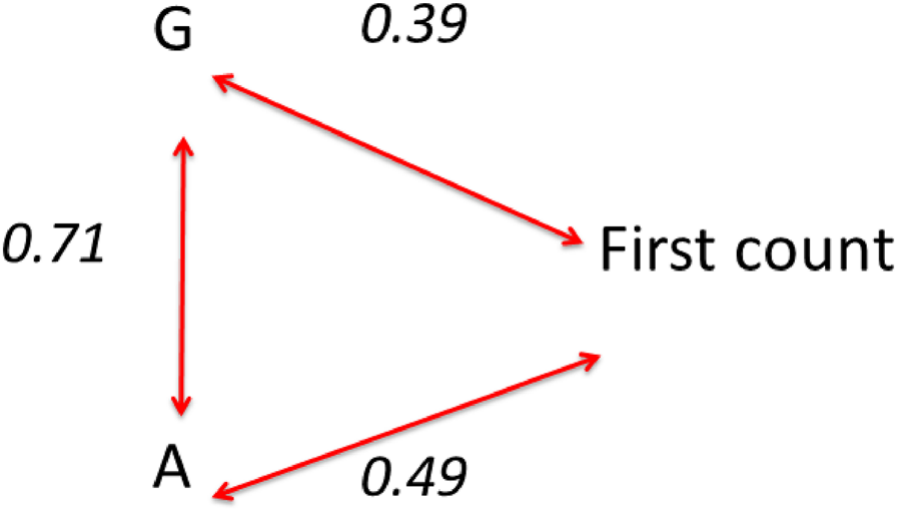
**Path analysis of allelic and genotypic richness.** Path analysis showing the direct effects, equivalent to correlation coefficients, of allelic richness (A) and genotypic richness (G) on the resistance to perturbation (as the number of shoots having survived the perturbation). The coefficient linking A and G is the correlation coefficient between these two components of genetic diversity. Path coefficients calculated after Alvin and Hauser (1975), and all are supported by p-values < 0.05.

Finally, path analysis confirmed that the strong correlation between A and G inflated the apparent effect of G on seagrass resistance to stress due to the hidden effect of changes in A with increasing G (Figure 2). Indeed, allelic richness had a stronger effect on resistance to disturbance, directly accounting for 25% of the variance (0.492) on the number of surviving shoots following stress, stronger therefore than genotypic diversity, which explained 15% of the variance (0.392; Figure 4). No significant effect was detected however on resilience, i.e. on shoot numbers 10 months after the algal bloom.

## Discussion

### Confounding effects of genotypic and allelic richness

This study makes a first attempt to test for the effect and interaction of allelic richness, a component of genetic diversity that has not been manipulated in previous experimental studies. The first important result is the relationship between genotypic and allelic richness at the low levels of genotypic richness typically used thus far in similar experiments (Figure 1). Indeed, results reported here show that it is unrealistic to dissociate the effect of allelic and genotypic richness on the whole experimental setup. Their high correlation prevented the setup of standardized levels of allelic richness for the three levels of genotypic richness used here (Figure 1b) and equal or equivalent to those manipulated in previous and similar studies. The simulation of 1000 random combinations of allelic richness for each level of genotypic richness showed a clear correlation between these two parameters (r = 0.904, p < 0.001) that precludes the dissociation of their respective effects in a regular two-way ANOVA. Such a strong correlation is particularly expected when allelic richness levels are ignored in an experimental design, or in the absence of any deliberate attempt to reach comparable levels across different genotypic richness. Should these parameters show such high correlation in the wild, each one could reflect the other, and be an equivalent *proxy* for the resistance or resilience of populations, thereby supporting the use of genotypic diversity emphasized in previous experimental studies. However, this extreme level of correlation is only valid at very low levels of genotypic richness such as those typically used in manipulative experiments ([60]; Figure 1a). The increase of allelic richness together with genotypic richness reaches a plateau soon after 20 MLG at levels of genotypic richness more commonly found in the wild (Figure 1b). This confirms recent and similar results based on both simulation and observational data on a terrestrial grass species [60], and our results on several meadows of other seagrasses (*Zostera marina* and *Posidonia oceanica*) which show that a correlation between allelic and genotypic richnesses also becomes at best marginal above levels of genotypic richness lower or similar as those commonly observed in natural meadows. It appears therefore essential to discriminate their respective effects in order to advance towards unravelling the underlying mechanisms responsible for differential survival, and not to erroneously interpret GR as a good *proxy* for AR (or the other way round) as their entanglement does not appear systematic in the wild.

In agreement with multiple regression results, path analysis supports a stronger effect of allelic richness on resistance to disturbance (25% of the variance on the number of surviving shoots following stress) than genotypic diversity (15% of the variance; Figure 4). Hence, the apparent direct effect of genotypic diversity on the number of surviving shoots following stress (Figure 2) is likely to be dominated by an indirect relationship with allelic richness (Figure 4). Hidden treatments remain a major pitfall of experimental studies testing the relationship between ecosystem functions and biodiversity, particularly those involving species richness [65]. Huston [65] defines “hidden treatments” as the situation arising where an experimental manipulation has multiple components, but only one of them is identified as the experimental treatment. Under this situation, erroneous conclusions about cause and effect relationships are likely because the actual cause of any observed response may be ignored in the interpretation of the experimental results, which can be considered a “hidden treatment.” Indeed, the results presented here show that allelic richness is a likely hidden treatment in previous experimental tests concluding on a positive role of genotypic diversity on seagrass resistance [50,66] or resilience [51,57] to perturbations. The lack of relationship between genotypic richness and heterozygosity was tested for in some cases to ensure that any observed effect was due to genotypic richness (i.e. clonal richness) rather than heterozygosity as a measure of genetic diversity *sensu stricto*[51], but the level of allelic richness, a more accurate indicator of the evolutionary potential of a population [61,62], was not mentioned in these studies. The consequences of ignoring allelic richness as a component of genetic diversity is that the strength of the effects assigned to genotypic diversity is inflated due to the unavoidable correlation between genotypic and allelic diversity in experimental tests. In fact, overlooking the allelic richness effect in our experiment, would also suggest enhanced resistance with increasing genotypic richness (Figure 2) similar to reports from previous studies [50,51,57]. However, the careful dissection of both parameters using path analyses allowed here to suggest a stronger direct effect of allelic richness than that of genotypic richness.

Our simulation analysis indicates that it is possible to dissociate effects of allelic and genotypic richness at much higher levels of genotypic richness than those typically used in experimental assessments. Indeed, the levels of genotypic richness that must be manipulated to allow separation of these effects are close to the levels recorded in natural meadows (up to 90% of sampling units for this species [67]). These levels are so high that they are hardly amenable to experimental test. When analysing shoot density at the only comparable levels of allelic richness, the only significant trends reflects a negative influence of G detected at A = 41. These results are in contradiction with previous reports on the positive effect of genotypic richness on resistance of experimental populations of *Zostera marina*[50,57,68] but in agreement with field surveys suggesting either the lack of effect or the negative influence of G *in situ* on *Posidonia oceanica*[64] and *Zostera marina* (Arnaud-Haond and Becheler, com. pers.). Experimental results reported here rather favour a positive effect of allelic richness on resistance of populations, while no clear trend or effect of genotypic richness could be inferred. Later after recovery, the interaction of allelic and genotypic richness seems to influence resilience of populations according to stepwise regression (but not path) analysis.

Hence, we submit that previous studies testing the role of genetic diversity on seagrass resistance [50,66] or resilience [51,57] to perturbations, using genotypic richness as a *proxy* for genetic richness may need be reassessed to test for the likelihood that allelic richness was a hidden treatment. In addition to accurately assigning effects to genotypic *versus* allelic richness, as done here using path analysis, one may bear in mind that allelic richness is an estimate of the number of genetic variants per locus meaningful for all organisms, whereas genotypic richness that reflects the proportion of genetically distinct individuals is only relevant for organisms capable of clonal propagation. The predominant influence of one or the other has therefore different interpretations in evolutionary terms, and major implications for management plans. Whether the decisive parameter for resistance or resilience is the number of distinct clonal lineages or the number of different genetic variants (alleles) they bear, or both, may lead to drastically different priorities and plans to protect, manage or even restore populations of clonal organisms. Future experiments should therefore systematically report both these components to avoid confounding effects, and should ideally attempt to separate both levels of allelic and genotypic richness, as also recently emphasized by Avolio et al. [60].

### Overall positive effect of genetic diversity on resistance to diatom invasion

This study supports a positive effect of intra-specific diversity on shoot survival immediately after perturbation. Algal blooms are increasingly reported and forecasted to intensify worldwide [69-72], often associated with coastal eutrophication, generating severe consequences particularly in terms of hypoxia and associated mortality events [73,74]. Global warming is expected to affect growth and life histories of diatoms [75-83], and several studies have already reported the negative effect of diatoms and other epiphytes on seagrasses [84-86], which may experience mortality due to suffocation by excessive growth of associated epiphytes and macroalgae. Diatoms grow on the leaves and may even fully cover them, preventing them from capturing light and eventually leading to the death of the shoots [66,87]. This experimental study provides a first record of the negative effect of diatom blooms on a key-species of coastal ecosystems, the impact of which can be buffered at higher levels of allelic and possibly genotypic richness. The percentage of shoot loss in the tank that suffered the algal diatom bloom exceeded 50% in all cases (65.23% ± 11.79%), revealing sub-lethal stress and mortality.

### The genetic component of diversity in the diversity-stability debate

The significance of the relationship between survival, measured 40 days after the bloom, and allelic richness supports, at least on seagrass experimental populations, two classical hypotheses underlying conservation genetics studies: i) the positive effect of the genetic component of diversity as estimated through allelic richness on resistance of populations, and ii) that a large enough set of neutral markers delivers reliable estimates of the level of polymorphism for the whole genome, including those genes potentially involved in a variety of responses to selective pressure [88,89]. No such tendency was detected on the resilience of experimental sub-plots after 10 months survey, but a positive effect of the interaction between allelic and genotypic richness was suggested.

Biodiversity has been shown to enhance the ecosystem’s ability to cope with stress [90] but ecosystems strongly depending on one key habitat-forming species, as is the case for intertidal meadows of *Z. noltii* in the Ria Formosa, may therefore be particularly dependent on the genetic diversity of these structural species.

Ecosystems may contain functional redundancy whenever species are capable of replacing each other [11-13,91]. While some species may decrease their contribution to ecosystem functions in face of environmental changes others would follow an opposite trajectory, thereby compensating losses [92-94]. The functional redundancy hypothesis can also be transposed to the level of genotypic and genetic diversity, especially when structural species are concerned. Hence, higher clonal (i.e. genotypic richness) and/or genetic (allelic richness) diversity may also be expected to increase the likelihood that the population will display a broader range of responses to variable conditions, displaying a higher phenotypic diversity for key traits as demonstrated in terrestrial plants [60], and therefore higher population stability. In the same way as each species in the community contributes with its unique use of resources and response to perturbations, different sets of alleles that may seem functionally redundant under some circumstances may fill different roles under changing conditions [50].

Hypotheses based on interaction among species, such as synergy or facilitation effects, can be similarly transposed to genotypic and allelic richness. This is particularly so for those based on observation of the importance of phylogenetic diversity in species assemblages, suggesting a higher contribution of distantly related species [11-13]. Mechanisms underlying the increased resistance of experimental assemblages observed here with higher A, and possibly G, cannot be unravelled with the experiment initially designed to explicitly consider A, not only G as in previous studies. Results suggest however several possible and non mutually exclusive interpretations that, although speculative, may contribute to feed future experiments or surveys to sort out these mechanisms. Previous studies on artificial assemblages concluded that the positive effect of genotypic richness could possibly have been due to the “insurance hypothesis”, suggesting that diverse assemblages are more likely to include genotypes able to resist particular environmental conditions, or “facilitation”, with more diverse assemblages showing higher complementarity in resource use [51,57]. Similar interpretations could be made when reasoning on the basis of allelic instead of genotypic richness. While it may be expected that interaction among genotypes (competition, facilitation or synergistic effects) may be enhanced at small scale, the differential adaptive capacity to distinct environmental conditions provided by distinct alleles or sets of alleles (genotypes) may be expected to influence resistance and resilience of natural populations at larger scales.

At the spatial and diversity scales explored in this and previous experiments, the effect of specific genotypes and of their interaction in a homogeneously impacted environment is expected to be favoured. At this scale, both genotypic and genetic diversity as estimated through allelic richness may lead to higher redundancy, as well as increased complementarity, synergistic or sampling effects. The results obtained here indeed suggest that assemblages showing higher A, and therefore bearing genotypes with more divergent genetic backgrounds are showing higher survival rates. Such observation at intra specific level also recalls the reports on the positive influence of phylogenetic divergence in species assemblages [12,13], suggesting facilitation or synergistic effect with assemblages of more distinct genotypes experiencing an enhanced complementarity in resources use. This is however contradictory with recent observations on *Z. marina,* suggesting a higher survival of assemblages composed of more genetically related genotypes [10]. Alternatively, the increased survival of assemblages with higher A observed here may be due to a sampling effect with genomic backgrounds with increased metabolic efficiency under stressful conditions met during the experiment, more likely to be present in more diverse assemblages. A beneficial effect of decreased competition among genotypes at small spatial scale, if existing as suggested by the apparent negative effect of genotypic diversity at intermediate levels of allelic richness, would favor an optimal trade-off made of decreased genotypic and increased genetic polymorphism as estimated through allelic richness (congruent with either sampling or niche differentiation effects; [95]). Finally, it may also be speculated that the interaction between allelic and genotypic richness seemingly influencing resilience may be due to the small spatial scale of experiments leading to an increased effect of inter-clonal competition through time. The relatively stabilized environmental conditions at the end of the algal bloom allowed experimental assemblages to evolve toward an equilibrium situation, possibly shifting the dominant force underlying survival and growth from differential adaptive capacities in stressful conditions, to competition for resources in a stabilized environment.

In terms of implications for conservation genetics, our results support the correlation between intra specific diversity and resistance or resilience by previous studies [50,57,66]. Yet they also suggest that these previous interpretations of the importance of genotypic (i.e. clonal) richness may be attributable to a hidden treatment, that of increasing allelic richness, which potential influence should therefore not be overlooked in conservation and restoration plans.

## Conclusions

This study reports the first experimental manipulation of both genotypic and allelic richness in a structural species, in an attempt to dissociate the effect of both parameters on its demographic response to stress. Our study shows that the two effects can hardly be disentangled at low levels of genotypic richness and that allelic richness may have been a hidden treatment in previous experiments reporting the positive effect of genotypic diversity on resistance and resilience of experimental populations. Results are in agreement with a positive effect of allelic richness on the resistance of populations to environmental stress, and suggest a positive effect of the interaction between genotypic and allelic richnesses on their resilience. This study underlines the potential importance of genetic diversity for the persistence of populations, an issue of particularly great concern when affecting key-species of an ecosystem such as seagrasses, and strongly indicates the importance of taking this parameter into account in management strategies.

## Methods

Synthetic assemblages of *Zostera noltii* shoots were setup in order to test for the existence of a relationship between allelic and/or genotypic richness and the resistance or resilience to stress conditions. Nine experimental treatments (= 9 subplots) were planned, each replicated 4 times (= 4 plots, Figure 1a), corresponding to the crossing of three levels of genotypic richness (3, 6 and 9 genotypes) with three levels of allelic richness (low, medium and high, for each level of genotypic richness). These 9 crossed levels where defined after *a priori* genotyping of a large number of shoots collected the field, and examination of the possible combinations of genotypic and allelic richness from those samples (Table 2, Figure 1). Below we provide the details for each step of this process up to the setup of each plot of nine subplots with increasing allelic and genotypic richness, resulting in a total of 4 plots each including 36 subplots.

### Sample preparation

A total of 376 plants with at least 10 shoots connected by one rhizome were collected in March 2009 from a single natural meadow in the Ria Formosa, Portugal, at the channel of Ramalhete in a restricted area of approximately 2 km^2^. Plants were then acclimated in a tank with running seawater and simulated tides for approximately 2 weeks. Each plant was tagged and one or two shoots used for DNA extraction using a standard CTAB extraction procedure [96] and genotyped for 9 microsatellite markers [67,97].

### Pre-selection of genotypes for the experimental setup

Among the list of 376 genotypes obtained, the distinct genets were recognized based on their multi locus genotypes (MLGs) assessed with the 9 microsatellite markers following [58]. The multi-locus genotypes were then used to virtually generate one thousand combinations of multi-locus genotypes (Figure 1b, c) for each of three genotypic richness levels (3, 6 and 9 MLGs) using a version of GenClone computer routine modified for that purpose ([98], available on request). The thousand combinations obtained for each level of genotypic richness were then sorted for their levels of allelic richness. Frequency distributions of allelic richness levels were drawn for the combinations generated for each genotypic richness level (Figure 1b, c). The ability to standardize allelic richness (as total number of alleles for all loci) for each level of genotypic richness was explored, and three values of A were selected to correspond to minimum, medium and maximum levels (Table 2). One combination of 27 shoots corresponding to each pair of genotypic and allelic richness levels was then selected to set each subplot.

### Experimental setup

Each subplot was set up in an individual small vase (approx. 2.5 L) with sediment from the Ria Formosa with a standardized initial density of 27 shoots per vase, comprising 9 sets of 3 connected shoots each. In order to respect a perfectly standardized setup in terms of number of shoots and connections, subplots with a genotypic richness of 3 MLGs had 3 sets of 3 shoots from each one of the 3 genets; subplots with a genotypic richness of 6 MLGs had a mixture of 3 shoots from 3 genets and 6 shoots from the other 3 genets split in two fragment of 3 shoots; and subplots with a genotypic richness with 9 MLGs had sets of 3 shoots from each one of the 9 different genets (Table 2; Figure 1d). Four replicates of 9 experimental treatments formed a total of 36 sub-plots (i.e., 36 vases with 27 shoots each, which were randomly distributed within a single aquaculture tank of approximately 1 m^3^). In order to make experimental conditions as close as possible from field ones, the tanks were filled with running seawater pumped from the Ria Formosa and a system was installed to mimic tide effects at a timing comparable to the natural periodicity.

### Stress treatment

Algal bloom was the stress treatment in this work, although it had been initially designed for a temperature shock. The temperature shock was not applied because the tank where all the experimental plots were set suffered a diatom bloom two weeks after the assemblage of the combinations, leading to significant seagrass mortality. The diatoms formed an epiphytic layer over the leaves. Mortality reached a sub-lethal level in this tank, whereas no comparable loss was recorded in two other neighbouring tanks where other unrelated experiments with *Z. noltii* had been setup with the same tide and water conditions. This supports the role of the diatom bloom, unique to this tank, as responsible for the sublethal stress and partial mortality. The resistance of the plants was monitored after the end of the bloom, approximately 40 days later, by counting the remaining shoots in each subplot. Resilience, as the capacity to recover, was estimated following the same parameter every two weeks for ten months after the algal bloom (data not shown).

### Statistical analysis

The effect of genotypic richness was initially meant to be assessed by two-way ANOVA (Figure 1a). Yet the impossibility to set subplots with standardized levels of allelic richness for each of the three levels of genotypic diversity explored (see results section; Figure 1d) limited the pertinence of this analysis. It was indeed impossible to disentangle the effect of allelic richness that remained partly hidden in the genotypic richness level in a standard two-way ANOVA (i.e. increasing levels of genotypic richness were associated to a parallel increase in the levels of allelic richness; Figure 1b).

In order to illustrate results expected when controlling and measuring only one of those two parameters and leaving the other as a hidden treatment, regressions were also performed on the three levels of genotypic richness ignoring allelic richness differences, and the effect of allelic richness was assessed by regression for each genotypic richness treatment.

In order to attempt discriminating the effect of each parameter, we proceeded as follows.

At a standardized level of allelic richness A = 31, the only level of A represented in all three genotypic richness levels, we assessed genotypic richness effects by a one-way ANOVA on shoot density at the first count after the algal bloom (i.e. resistance) and at the last count taken eleven months later (i.e. resilience). This level of allelic richness was found in plots exhibiting the maximum levels of Â richness for 3 MLG, medium for 6 and minimum for 9 (Figure 1d). Similarly *t*-test where performed to compare only two means of density, for A = 25 (an allelic richness level common to plots with medium levels of allelic richness for 3 MLG and minimum for 6 MLG; Figure 1d) and A = 41 (an allelic richness level common to plots with maximum allelic richness for 6 and medium for 9 MLG; Figure 1d). The effect of allelic richness was also assessed by one-way ANOVA on shoot density performed at each genotypic richness level individually, both on data of resistance collected right after mortality linked to the algal bloom and at the end of the resilience survey.

The overall role of allelic and genotypic richness in the performance of the plants along the various stages of the experiment was evaluated through multiple linear regressions by evaluating the model: Y = b0 + b1R + b2A + b3R*A using a stepwise backward procedure. ANOVA and regressions were performed using STATISTICA (STATISTICA 7.0, StatSoft, Inc.). A correction for multiple tests described here above and compiled in Table 3 was performed using the q-value method for estimating the false discovery rates (FDR) from the distribution of P-values. A P-value was considered significant at the 5% level when the qvalue was <5% [99]. Finally, in order to account for the strong correlation between G and A, we used path analysis to dissociate the indirect (due to correlation between components of genetic diversity) and direct effects of genotypic and allelic richness. Path analysis is a statistical tool specifically designed to separate direct from indirect effects of closely correlated independent variables on dependent variables [100]. By evaluating the direct and undirect components of (statistical) effects, path analysis implicitly evaluate possible alternatives, but applied here on a simple system with only two variables simply yields the optimal result for their covariance structure.

## Authors’ contributions

SIM conducted the experimental work, the data analyses and drafting of the manuscript. CMP helped with the sampling, genotyping and shoot density monitoring. CMD participated in the discussion of the findings and reviewed the manuscript. EAS and SAH were involved in the conception and design of this study, participated in the data analysis and reviewed the manuscript for important intellectual content. All authors read and approved the final manuscript.

## Authors’ information

Sónia Massa is a PhD student interested in molecular ecology and gene expression, with special emphasis on the diversity-stability debate. Cristina Paulino is a MSc student interested in the ecology of marine organisms. Ester Serrão leads a research group that is primarily interested in marine ecology, adaptation and population genetics. Carlos M. Duarte leads a team studying marine biodiversity from the genetic, species and habitat level to global biogeochemical cycles. Sophie Arnaud-Haond is an evolutionary ecologist interested in the factors driving the evolution of marine populations, with a special emphasis on the mating system and connectivity patterns.
